# Cascade screening for β-thalassemia: A practical approach for identifying and counseling carriers in India

**DOI:** 10.4103/0970-0218.58399

**Published:** 2009-10

**Authors:** Ajit C Gorakshakar, Roshan B Colah

**Affiliations:** National Institute of Immunohaematology (ICMR), KEM Hospital Campus, Parel, Mumbai - 400 601, India

## Introduction

β- thalassemia is one of the most common single gene disorders in India with an overall prevalence of 3-4%.(1) In certain communities like Sindhis, Muslims, Cutchi Bhanushalis, and some tribal groups, the prevalence of β thalassemia carriers varies between 8 and 10% or more.(2,3) It has been estimated that about 10 000-12 000 children with β thalassemia major are born every year in India. These figures might be underestimated.

The epidemiology of thalassemia is changing during the past few decades in some countries such as Cyprus and Greece. This is mainly due to the successful implementation of prevention programs. A reduction in the birth rate of babies with thalassemia major from 1:250 to 1:4000 over the years has been reported in Sardinia.(4)

India is a vast country where over 4000 ethnic groups with diverse cultural backgrounds are residing. Several communities have not been screened so far, and what is required is micro mapping at least at the district level to get an accurate estimate of the β–thalassemia gene in the population.

The individuals who are at an increased risk have some knowledge of thalassemia as compared to the general population where majority of the people are ignorant of this disorder. This is the first report on cascade screening for β-thalassemia from India.

## Materials and Methods

The parents of children with β-thalassemia major receiving blood transfusions regularly at various centers in Mumbai City in western India were contacted and after explaining the need to screen extended family members, the addresses of their close relatives(paternal and maternal family members over at least two generations) were obtained. A social worker then contacted these families. Counseling was given on the possibility of other family members being carriers and after getting an informed consent, blood samples were collected in EDTA. Red cell indices were measured on a well calibrated cell counter (ERMA PC 608) with controls being run regularly while Hb A_2_ estimation was done by cellulose acetate electrophoresis (pH 8.9) and elution.

## Results

**Figure 1:**
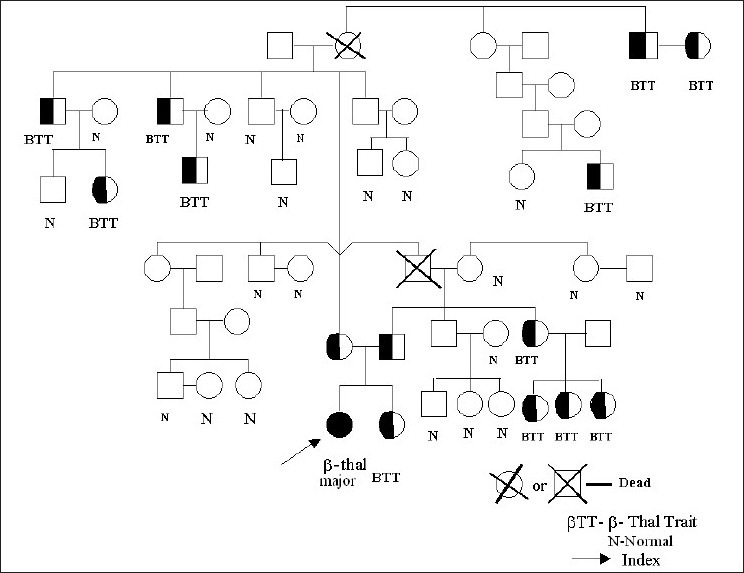
A typical pedigree of an index case

Majority of the affected children (index cases) were from “high risk” communities and 44 families were screened. (Sindhis -21, Jains -11, Cutchis -8, Muslims -2, Vaishnavas -2). Among these, 25 siblings of index cases were also screened, and 10 of them were β-thalassemia heterozygotes. Similarly, 490 children from high-risk communities were screened and 96 were β–thalassemia heterozygotes. In all, 151 of the 691 individuals screened were β-thalassemia carriers (21.9%). Among the normals (age range 5- 65 years), males had a mean Hb of 13.97 ± 1.51 g/dl while females had a mean Hb of 12.16 ± 1.74 g/dl while among β-thalassemia carriers (age range 3–58 years) males had a mean Hb of 11.96 ± 1.74 g/dl while females had a mean Hb of 10.86 ± 1.41 g/dl. Table 1 shows complete hematological data of β–thalassemia carriers and normal individuals detected during this study. A typical family pedigree is shown in Figure 1 where 18 young individuals were screened and seven of them were identified as β-thalassemia carriers premaritally.

**Table 1:** Hematological data (Mean ± SD) of β thalassemia carriers and normal individuals detected during cascade screening

Group	RBC (×10^6^/ μl)	Hb (g/dl)	HCT (%)	MCV (fl)	MCH (pg)	MCHC (g/dl)	HbA2 (%)	Hb F (%)
β -thalassemia carriers	5.7 ± 0.77	11.60 ± 1.75	36.56 ± 5.09	62.84 ± 5.82	19.92 ± 2.28	31.54 ± 3.32	4.99 ± 0.64	1.2 ± 0.42
Normal individuals	5.00 ± 2.97	13.03 ± 2.13	39.44 ± 5.80	81.23 ± 8.04	26.98 ± 3.38	32.97 ± 2.95	2.59 ± 0.45	1.0 ± 0.24

## Discussion

Carrier screening programs are being conducted in several countries using various approaches. They include screening of the general population, screening of ‘high-risk’ communities, antenatal screening, and cascade screening.

As compared with other approaches, the percentage of β-thalassemia carriers identified was 5-6 times higher using this cascade screening approach. It has been shown earlier that in communities where consanguinity is common, one can identify even more number of carriers, for example, 31% carriers were identified during cascade screening in Pakistan.(5) In a small place like Sardinia, the outcome of cascade screening was more impressive. More than 90% of the “at risk” couples were identified by examining only 11% of the population.(6) Similarly, Super *et al*.(7) found that cascade screening for cystic fibrosis was ten times more efficient in detecting carrier couples as compared to unfocussed screening. The large size of families as well as the system of living in joint families rather than nuclear ones is an advantage in this approach. Identification of unmarried carriers among the relatives of the index cases is very important as it gives them the option of selecting a partner who is a non–carrier. Alternatively, they have the option of prenatal diagnosis if the partner is not tested premaritally and the couple is found to be ‘at risk’.

In 1990, Sangani *et al.*(8) collected information on the attitudes toward screening for thalassemia among 200 families of index cases residing around Mumbai. Twenty percent of these families had expressed an unfavorable reaction from their relatives. Hence, these parents were secretive about their child having thalassemia major to avoid social stigmatization. Yagnik(9) followed up 70 carriers and 127 non-carrier who are identified during screening of high-risk communities 5–7 years after counseling. Of these, 46% of the carriers had got all their siblings tested while 42% had not informed their siblings about their carrier status. Recently, information collected from parents of 100 index cases from North India showed that 96% of the parents of affected children were ready to share information on thalassemia with their relatives.(10) This shows that after a decade, there has been a change in the attitude of parents and relatives of index cases in the understanding of an inherited disorder such as thalassemia. This may be due to the awareness generated in the population over the years.

In the questionnaire-based study from North India, the family members of 14% of the affected children could not get themselves tested due to non–availability of facilities for screening in nearby towns and also due to non–affordability.(10) Such a situation must be avoided by the establishment of more centers and cost-effective technology for screening. At the same time, doctors in Government hospitals as well as in private practice in rural areas should be trained to identify cases of β thalassemia major. Blood banks in these areas should be strengthened for adequate transfusion facilities for babies with thalassemia major.

Several non-government organizations (NGOs) and other agencies identify carriers, targeting mainly school and college students. Our experience suggests that screening of school children does not have the desired impact.(11) Screening of pregnant women in antenatal clinics is feasible; however, even in urban areas, only 10–15% of women come to antenatal clinics in the first trimester of pregnancy.(12) In rural areas many deliveries still take place at home. Relatives of children with thalassemia major have seen the stress on the family with an affected child and would therefore come forward more easily for carrier screening. Therefore, once the index case is identified; cascade screening seems to be cost effective. In rural areas, many times close relatives live in the same village making screening and counseling more acceptable. Endogamy and consanguinity are still important features of our population. Owing to this, it is likely that pockets with high prevalence of a recessive character such as β-thalassemia may exist in areas where screening has not yet been done.

## Conclusions

A national control program for thalassemia has not yet been formulated in India. The important step in this program is the identification and counseling of carriers, and a combination of different approaches is required for its establishment.

After screening 691 extended family members, as many as 151 carriers were identified. We would not have identified as many carriers after screening 691 individuals, either the school children or antenatal women. On the basis of the prevalence of β–thalassemia carriers reported by us,(1,12) we would have had to screen about 5600 school children or 9500 antenatal women to identify 151 carriers. Further, minimal efforts were required to create awareness and for counseling this group of individuals. Therefore, this seems to be one of the most cost-effective and practical approaches to identify beta thalassemia carriers.

India has one of the largest private health care sectors in the world. Using public –private partnership, a good network must be developed for optimum care of children with β thalassemia major and for education and screening for the identification of carriers in a cost-effective way where cascade screening will have a significant role not only in big cities but also in small cities and rural areas in India.
